# Progression to active TB post-TB preventive treatment among household contacts in India: Axshya Plus project (2021–2024)

**DOI:** 10.5588/ijtldopen.26.0177

**Published:** 2026-07-13

**Authors:** B. Kalottee, A. Nuken, A. Dubey, P. Mahajan, H. Solanki, R. Taralekar, M. Yassin, N. Restrepo, R. Rao, S.K. Mattoo, V. Dhawan, S. Rajan

**Affiliations:** 1 International Union Against Tuberculosis and Lung Disease, India Office, New Delhi, India;; 2 World Health Organization (WHO), Country Office for India, New Delhi, India;; 3 Global Fund to Fight AIDS, Tuberculosis and Malaria, Geneva, Switzerland;; 4 Central TB Division, Directorate General of Health Services, Ministry of Health and Family Welfare; New Delhi, India.

**Keywords:** tuberculosis, TPT, HHCs, diagnosis

Dear Editor,

For high-risk groups, the WHO End TB Strategy emphasises early detection of TB along with expanded identification of TB infection (TBI) and provision of TB preventive treatment (TPT).^1^ Household contacts (HHCs) of pulmonary TB (PTB) patients are at risk due to proximity and prolonged exposure, high household transmission, variable immunity, and delays in diagnosis and treatment.^2^ In India and other high-burden settings, TB control efforts have historically prioritised treatment of active TB, with limited focus on TBI due to diagnostic and resource constraints.^3,4^ However, recent global commitments, including the United Nations High-Level Meeting (UNHLM) on Tuberculosis, and national policies under the National Tuberculosis Elimination Programme, reinforced scale-up of TPT including shorter regimens such as 3HP.^1,5,6^ Despite this progress, evidence gaps remain regarding post-TPT outcomes, particularly progression to active TB or drug-resistant TB (DR-TB). Initiation of TPT without adequate exclusion of active TB may result in inadvertent monotherapy and drug resistance.^7,8^ National guidelines recommend periodic follow-up of HHCs post-TPT; however, long-term outcome data are limited.^4^

This study followed up HHCs post-TPT to assess progression to active TB or DR-TB and compare outcomes across regimens and programmatic approaches, including the value of diagnostic-guided ‘Test & Treat’ strategies.

We conducted a retrospective cohort study with cross-sectional outcome ascertainment of HHCs initiated on TPT (October to December 2021) under the Axshya Plus project across 62 districts in 7 Indian states, with outcomes assessed 2 years post-TPT. Implemented by The Union with Global Fund support (2021–2024), the project focused on programmatic TPT delivery among HHCs of PTB patients. Two programmatic approaches were used: ‘Test & Treat’, where HHCs were tested for TBI (interferon-gamma release assay/tuberculin skin test) and if positive, offered TPT; and ‘Treat Only’, where TPT was provided after ruling out active TB without TBI testing. Eligible HHCs received either 6H (daily isoniazid for 6 months) or 3HP (weekly isoniazid and rifapentine for 3 months). TPT adherence was monitored through pill counts, in which remaining doses were counted at follow-up visits, and documented systematically, complemented by routine programmatic follow-up, in line with national PMTPT guidelines.^4^ A representative sample of 2,519 HHCs was followed up post-TPT (state-level random sampling or census for smaller states). Data were collected by Team Leads/TBI Coordinators using structured questionnaires, verified by state programme managers or technical advisors, and rechecked via telephonic follow-up. Categorical variables were summarised as frequencies/percentages, and continuous variables as median (interquartile range [IQR]). Post-TPT progression to active TB/DR-TB was assessed across regimens and programme approaches, and confidentiality was maintained. As no additional intervention or identifiable data were collected beyond routine programme activities, formal ethical approval was not obtained.

Among 2,507 HHCs followed up post-TPT, 2,404 received 6H (2,188 Treat Only; 216 Test & Treat) and 103 received 3HP (89 Treat Only; 14 Test & Treat). Median age was 31 years (IQR 16–45); 54% were female. Age-stratified outcomes showed that children 5–14 years had a slightly higher progression rate to active TB (3/268; 1.12%) compared with those <5 years (1/316; 0.32%) and ≥15 years (13/1,923; 0.68%). Seventeen HHCs (0.68%) developed active TB post-TPT. Among these, 13 had PTB, 3 had extra-pulmonary TB (all <18 years), and 1 DR-TB (6H recipient). Median time to progression was 10 months (IQR 7–13), shorter in women (7 months) than men (10 months). Sixteen cases were in the 6H group, and only one in 3HP. Importantly, all cases occurred in the Treat Only group, with none in Test & Treat. Geographically, cases were reported in all states except Chhattisgarh and Himachal Pradesh, with the highest in Maharashtra (9/17) (see Table 1).

**Table.** Characteristics of household contacts and active TB cases post-TB preventive treatment (TPT).

	Total interviewed	Active TB cases (in number)	Active TB cases (%)
Age groups			
Less than 5 years	316	1	0.32
5–14 years	268	3	1.12
15 and above years	1,923	13	0.68
Gender			
Men	1,152	8	0.69
Women	1,355	9	0.66
Type of TPT Regimen			
6H	2,404	16	0.67
3HP	103	1	0.97
TPT model			
Treat only	2,277	17	0.75
Test and Treat	230	0	0.00
TPT regimen & TPT model			
3HP & Treat only	89	1	1.12
3HP & Test and Treat	14	0	0.00
6H & Treat only	2,188	16	0.73
6H & Test and Treat	216	0	0.00
State			
Assam	138	1	0.72
Chhattisgarh	115	0	0.00
Himachal Pradesh	252	0	0.00
Jharkhand	500	4	0.80
Madhya Pradesh	500	1	0.20
Maharashtra	501	9	1.80
West Bengal	501	2	0.40
Total	2,507	17	0.68

The low post-TPT progression rate (0.68%) underscores TPT’s effectiveness in preventing progression to active TB among HHCs, substantially lower than the estimated 5%–15% lifetime risk among individuals with TBI,^2,9^ often within 2 years of exposure, with reported progression risks of <1%–19.7% during and/or after TPT,^10,11^ highlighting TPT’s protective effect and the need for ongoing monitoring. Adherence monitoring through pill counts and regular follow-up (in-person and telephonic) strengthens confidence in this finding, as adherence is a key determinant of TPT effectiveness and treatment completion is critical for preventing progression to active TB.^2^ Progression was slightly higher in HHCs aged 5–14 years (1.12%) compared with <5 years (0.32%) and ≥15 years (0.68%), highlighting the need for focused follow-up in this age group. Post-TPT progression was similar between genders, although women progressed slightly earlier (median 7 vs. 10 months), potentially reflecting biological, behavioural, or contextual factors. By regimen, progression occurred within 0–15 months among those receiving 6H, while the single case in the 3HP regimen group occurred at 19 months post-TPT. Although limited by singular event, this variation may suggest a longer duration of protection with 3HP. This observation should be interpreted cautiously but may inform regimen selection, particularly regarding durability of preventive effect. While broadly aligned with global recommendations favouring shorter, rifamycin-based TPT regimens,^2,7^ further studies are needed to confirm the long-term protective effects of the 3HP, especially in high-burden settings where both efficacy and adherence are critical.

All 17 active TB cases occurred in HHCs in the Treat Only model (16 with 6H and 1 with 3HP), where mandatory TBI testing was not performed prior to TPT initiation. No cases were observed in the Test & Treat group, suggesting that diagnostic-guided TPT may offer additional protection against progression and underscores the importance of pre-treatment screening to exclude active TB and avoid inadvertent monotherapy and DR-TB. Geographical clustering in Maharashtra and Jharkhand may reflect differences in TB exposure, follow-up practices, or programmatic coverage. Given limited TBI testing resources, the Treat Only model may provide a pragmatic safety net, though its effectiveness is limited and not foolproof, even with mandatory chest X-ray. Reluctance to visit testing centres due to wage loss, socio-economic and cultural barriers, and operational or diagnostic challenges continues to limit the success of the Test & Treat model.^12–14^

Limitations of our study include cross-sectional follow-up, a small number of post-TPT TB cases limiting statistical power, potential confounding from undiagnosed baseline TB, adherence, limited follow-up duration, and unmeasured factors such as co-morbidities and socio-economic conditions. Additionally, differences in geographic and programmatic coverage may influence observed outcomes. Nonetheless, these findings provide programmatic insights supporting scale-up of TPT, particularly diagnostic-guided approaches, to prevent progression to active TB and reduce DR-TB risk.

Post-TPT progression to active TB among HHCs was rare, and no cases occurred in the Test & Treat group at 2-year follow-up. These findings support the protective effect of TPT and highlight the value of diagnostic-guided strategies, particularly in high-burden settings. Younger contacts and Treat Only districts may benefit from intensified monitoring and targeted interventions. Overall, TPT remains a cornerstone of TB prevention, with programmatic implications for regimen selection and follow-up strategies.
